# American Medical Association—Section of Dental Surgery

**Published:** 1892-05

**Authors:** 


### American Medical Association--Section Dental Surgery.

The following named gentlemen have accepted the invitation to read papers before the Dental Section of the American Medical Association, to be held at Detroit, in June, viz.: Dr. G. S. Junkerman, Cincinnati, O.; Dr. T. D. Crothers, Hartford Conn.; Dr. J. Smith Dodge, New York City; Dr. W. C. Barrett, Buffalo, N. Y.; Dr. Briggs, Boston, Mass.; Dr. Whitefield, Evanston, Ills.; Dr. J. L. Gish, Jackson, Mich.; Dr. J. S. Marshall, Chicago, Ills.; Dr. M. H. Fletcher, Cincinnati, O.; Dr. H. Gadle, Chicago, Ills,; Dr. G. W Weld, New York City; Dr. G. L. Curtis, New York City and Dr. E. S. Talbot, Chicago, Ills. Still other papers are to come for that occasion; it is expected that this will be one of the largest meetings of this section ever held.

